# 合并未分化子宫肉瘤的成人间变性淋巴瘤激酶阳性组织细胞增生症1例

**DOI:** 10.3760/cma.j.cn121090-20251119-00538

**Published:** 2026-04

**Authors:** 佳琦 徐, 蓓蓓 吕, 文博 赵, 晓燕 林

**Affiliations:** 1 山东大学齐鲁医学院，济南 250012 Cheeloo College of Medicine, Shandong University, Jinan 250012, China; 2 山东第一医科大学附属省立医院（山东省立医院）病理科，济南 250021 Department of Pathology, Shandong Provincial Hospital Affiliated to Shandong First Medical University, Jinan 250021, China; 3 山东第一医科大学附属省立医院（山东省立医院）血液内科，济南 250021 Department of Hematology, Shandong Provincial Hospital Affiliated to Shandong First Medical University, Jinan 250021, China

患者，女，37岁，因“腹痛，阴道不规则流血”于2024年7月1日入我院。患者自述于1个月前月经期开始出现下腹隐痛，未就诊治疗。3日前出现大量阴道流血伴腹痛。1天前出现高热，最高体温39 °C。血常规：WBC 35.15×10^9^/L、RBC 2.92×10^12^/L、HGB 86 g/L、PLT 414×10^9^/L、嗜酸性粒细胞百分比40.3％、单核细胞绝对值0.81×10^9^/L、中性粒细胞绝对值17.26×10^9^/L、嗜酸性粒细胞绝对值14.17×10^9^/L、嗜碱性粒细胞绝对值0.25×10^9^/L。骨髓穿刺结果：骨髓增生明显活跃，三系细胞可见，粒红比例升高，嗜酸性粒细胞明显增多。腹盆腔CT平扫显示：双肺少许微结节；子宫壁多发大小不一低密度灶伴不均质强化，以子宫左后壁为著，部分区域外凸，边缘不清晰，伴部分区域低密度融合（短径约7.0 cm），宫腔略受压偏右，双侧附件轮廓境界欠清，与子宫分界欠清（左侧为著），盆腔见中量积液。盆腔内膀胱前上方，双侧髂血管周围、腹股沟区多发淋巴结肿大；右肾下级低密度灶。随后行腹膜后淋巴结穿刺活检。大体检查：穿刺组织3条，长0.5～1.0 cm，直径小于0.1 cm，部分断裂，灰白灰红。镜下检查：未见淋巴结结构，淋巴细胞少见，以短梭形细胞和卵圆形细胞为主，伴少量嗜酸性粒细胞浸润。免疫组织化学染色结果：CD68、CD163、Vimentin以及间变性淋巴瘤激酶（ALK）阳性，S-100和SMA弱阳性，Desmin和LCA少许细胞阳性，Ki-67阳性指数10％～30％，CD1a、Langerin、SOX10、STAT6、CgA、CK、HMB45、CD3、CD4、CD30、CD20、CD10、Cyclin D1、CD21、CD35、CD123、Dog-1、MPO和ER均为阴性表达。病理诊断：符合ALK阳性组织细胞增生症（APH）。分子检测：ALK基因分离探针FISH检测结果提示ALK基因断裂。经扩增子法联合二代测序（NGS）检测，本例患者样本明确检出CLTC基因31号外显子与ALK基因20号外显子融合突变，测序结果可清晰比对到双基因的断裂位点，证实该融合变异真实存在。

患者拒绝进一步检查和治疗自行出院，2024年7月15日于外院行宫腔活检，病理诊断倾向未分化子宫肉瘤。患者行退热等对症处理后，静脉化疗2个周期，具体用药：达卡巴嗪0.4 g（第1天到第4天）+顺铂40 mg（第1天至第3天）+多柔比星70 mg（第1天）。2024年8月29日患者行经腹筋膜外全子宫切除+双侧附件切除+大网膜切除+腹盆腔肿物切除+肠粘连松解术。术后病理检查，全子宫：梭形细胞恶性肿瘤，肿瘤组织伴大量坏死，部分肿瘤细胞退变，伴小灶钙化，符合未分化子宫肉瘤治疗后改变；肿瘤侵犯子宫肌壁全层，免疫组织化学染色检测：CD10部分区域阳性表达，Cyclin D1局灶阳性表达，Vimentin阳性表达，EMA散在阳性表达，SMA、Desmin、CKpan、CK7、CK8/18、ER、PR、MyoD1、Myogenin均为阴性表达，Ki-67热点区约60％。术后病理切片中未见明确APH成分。未分化子宫肉瘤分子检测：ALK、EGFR、PDGFRA、JAK2 FISH检测结果均为阴性。患者于2024年10月5日因子宫恶性肿瘤终末期合并电解质紊乱、恶病质，呼吸循环衰竭死亡。

